# Editorial: Use of Saliva in Diagnosis of Periodontitis: Cumulative Use of Bacterial and Host-Derived Biomarkers

**DOI:** 10.3389/fcimb.2016.00196

**Published:** 2016-12-22

**Authors:** Ulvi K. Gursoy, Eija Könönen

**Affiliations:** ^1^Department of Periodontology, Institute of Dentistry, University of TurkuTurku, Finland; ^2^Oral Health Care, Welfare DivisionTurku, Finland

**Keywords:** saliva, biomarker discovery, periodontal diseases, dentistry, oral health

Oral cavity is the gate of the gastrointestinal tract, where the digestion process begins. Due to a wide range of nutritional intake, the oral cavity is constantly prone to physical and chemical stress. Mouth with its different types of surfaces and a warm and humid atmosphere offers a comfortable environment for a myriad of microbes to survive and replicate easily. Oral microbiota is an essential part of the host-tissue homeostasis; it contributes to the innate immune response and nitric oxide synthesis (Sintim and Gürsoy, 2016). However, bacterial colonization on non-shedding tooth surfaces allows the formation and maturation of biofilms where bacterial cells are well-protected from various host response activities. When pathogenic bacteria reside in these complex microbial communities, it may result in dysbiotic biofilms that get benefit from inflammation. Two wide-spread diseases of the oral cavity, connected to dysbiosis, are tooth decay and periodontitis.

In periodontitis, a degradative process occurs in tooth-supporting tissues, i.e., gingiva, periodontal ligament, and alveolar bone. The disease is initiated by bacteria; however, the main reason behind the tissue degradation is uncontrolled immune response. It often proceeds without any symptoms and, therefore, patients may seek professional help “at the point of no return” and experience loss of teeth. It is estimated that about 10–15% of the adult populations suffer from advanced periodontitis (Petersen and Ogawa, 2012). The prevalence and severity of periodontitis is increased in individuals with low socioeconomic status, tobacco use, excessive alcohol consumption, and certain systemic diseases, such as diabetes mellitus. According to the available data, the economic impact of dental diseases was 442 billion US$ worldwide in 2010 (Listl et al., 2015). Out of that sum, 298 billion US$ was the direct treatment costs, and 144 billion US$ was the indirect costs due to dental diseases (Petersen and Ogawa, 2012).

On one hand, a dentist can readily detect periodontal disease with conventional methods using a periodontal probe and, in case of clinical signs of periodontitis, radiographs. On the other hand, this type of diagnostics is economically costly and time-consuming and even not feasible to perform in large-scale studies. To use resources in an economic way, a cost-effective method to identify people suffering from periodontal disease is warranted.

Analysis of saliva for setting periodontal diagnosis is not a new idea. Information and experience on salivary diagnostics has improved tremendously during the past two decades. Saliva as a diagnostic tool offers several advantages; for example, by analyzing an array of constituents present in saliva, it is possible to monitor the disease onset and progression. Moreover, sufficient quantities of saliva can be collected in a non-invasive and simple way. Nevertheless, there are also shortcomings in salivary diagnostics. As an example, periodontitis has exacerbation and remission periods and the fluctuation in disease progression affects the diagnostic power of the salivary biomarker selected. Furthermore, systemic diseases (e.g., diabetes) and conditions (e.g., pregnancy), and local factors aggravating the progression of periodontitis (e.g., smoking) affect the levels of salivary biomarkers. To overcome these limitations, it has been proposed that the simultaneous detection of bacterial and host-derived biomarkers in saliva improves considerably the accuracy of periodontal diagnosis (Gursoy et al., 2011; Sorsa et al., 2016).

The present issue with its research topic “Use of saliva in diagnosis of periodontitis: cumulative use of bacterial and host-derived biomarkers” is composed of nine articles with altogether 49 authors, aiming to bring new information and aspects on the combinational use of biomarkers. In their mini-review, Ji and Choi discussed the challenges of point-of-care diagnostics of periodontitis and proposed an organization, International Consortium for Biomarkers of Periodontitis, to be built. Combinational analyses of salivary concentrations of several bacterial and host inflammatory markers were tested in two independent studies where the authors successfully demonstrated that these types of combinational analyses bring additional power to detect periodontitis (Ebersole et al.; Salminen et al.). Aboodi et al. followed the changes at an early phase of periodontal disease, performing proteome analyses in an experimental gingivitis model. Use of oxidative stress markers present in saliva for detecting periodontitis was discussed in a review Tóthová et al., while the study by Zhang et al. indicated a dysregulated immune response of being associated with antioxidant capacity in periodontitis patients. A combination of salivary nitrite and nitrate levels was found as a potential biomarker for phenytoin-induced gingival overgrowth, but this was not applicable for other drugs Sukuroglu et al. The role of antimicrobial peptides, small cationic peptides, in innate immune response and their usability as potential salivary markers of periodontitis were discussed in a review by Güncü et al. Finally, systems biology approach was used, up to our knowledge for the first time, to define putative host-derived biomarkers of periodontitis in saliva Zeidán-Chuliá et al.

We hope that the information brought together by the authors of the present issue will encourage researchers in the field of salivary diagnostics to have a new perspective to overcome the current limitations. The development of sophisticated methods will bring laboratory to clinics and the public.

## Author contributions

All authors listed, have made substantial, direct and intellectual contribution to the work, and approved it for publication.

### Conflict of interest statement

The authors declare that the research was conducted in the absence of any commercial or financial relationships that could be construed as a potential conflict of interest.
